# A New Sentinel Surveillance System for Severe Influenza in England Shows a Shift in Age Distribution of Hospitalised Cases in the Post-Pandemic Period

**DOI:** 10.1371/journal.pone.0030279

**Published:** 2012-01-23

**Authors:** Shelly Bolotin, Richard Pebody, Peter J. White, James McMenamin, Luke Perera, Jonathan S. Nguyen-Van-Tam, Thomas Barlow, John M. Watson, for the UK Severe Influenza Surveillance System (USISS) Steering Group

**Affiliations:** 1 Health Protection Agency, London, United Kingdom; 2 MRC Centre for Outbreak Analysis and Modelling, Imperial College School of Public Health, London, United Kingdom; 3 Health Protection Scotland, NHS National Services, Glasgow, United Kingdom; 4 Department of Health, London, United Kingdom; 5 University of Nottingham, City Hospital, Nottingham, United Kingdom; 6 Department of Health, London, United Kingdom; 7 Public Health Wales, Cardiff, United Kingdom; 8 Public Health Agency, Belfast, United Kingdom; 9 Royal College of Physicians, London, United Kingdom; University of Hong Kong, Hong Kong

## Abstract

**Background:**

The World Health Organization and European Centre for Disease Prevention and Control have highlighted the importance of establishing systems to monitor severe influenza. Following the H1N1 (2009) influenza pandemic, a sentinel network of 23 Trusts, the UK Severe Influenza Surveillance System (USISS), was established to monitor hospitalisations due to confirmed seasonal influenza in England. This article presents the results of the first season of operation of USISS in 2010/11.

**Methodology/Principal Findings:**

A case was defined as a person hospitalised with confirmed influenza of any type. Weekly aggregate numbers of hospitalised influenza cases, broken down by flu type and level of care, were submitted by participating Trusts. Cases in 2010/11 were compared to cases during the 2009 pandemic in hospitals with available surveillance data for both time periods (n = 19). An unexpected resurgence in seasonal A/H1N1 (2009) influenza activity in England was observed in December 2010 with reports of severe disease. Reported cases over the period of 4 October 2010 to 13 February 2011 were mostly due to influenza A/H1N1 (2009). One thousand and seventy-one cases of influenza A/H1N1 (2009) occurred over this period compared to 409 at the same Trusts over the 2009/10 pandemic period (1 April 2009 to 6 January 2010). Median age of influenza A/H1N1 (2009) cases in 2010/11 was 35 years, compared with 20 years during the pandemic (p = <0.0001).

**Conclusions/Significance:**

The Health Protection Agency successfully established a sentinel surveillance system for severe influenza in 2010/11, detecting a rise in influenza cases mirroring other surveillance indicators. The data indicate an upward shift in the age-distribution of influenza A/H1N1 (2009) during the 2010/11 influenza season as compared to the 2009/10 pandemic. Systems to enable the ongoing surveillance of severe influenza will be a key component in understanding and responding to the evolving epidemiology of influenza in the post-pandemic era.

## Introduction

The World Health Organisation and European Centre for Disease Prevention and Control have highlighted the importance of establishing surveillance systems to monitor the epidemiology of severe influenza and severe acute respiratory disease [1], [2]. The UK Severe Influenza Surveillance System (USISS) is a web-based scheme established in 2010/11, which collects surveillance data on hospitalised influenza cases from a sentinel network of National Health Service (NHS) Acute Trusts (usually comprising of one or a group of hospitals) across England. The scheme aims to monitor the impact of influenza on the population and describe the epidemiology of severe influenza in time, place and person. Data collected through USISS aims to assist in the evaluation and development of clinical guidance as well as support the development of policy, and has been instrumental in determining the feasibility of a routine hospital surveillance system in England.

During the 2009 influenza pandemic, the UK experienced two waves of activity, punctuated by the school summer holidays, which affected primarily children and young adults. The second wave peaked in late October 2009 and declined by February 2010, after which little influenza activity was observed until late 2010. From early December 2010, the United Kingdom experienced a sharp increase in influenza activity, peaking in late December and then declining through January and into February 2011. Starting in early December, the start of the season was heralded by a rapid increase in reports of hospitalisations, intensive care admissions and fatalities due primarily to influenza A/H1N1 (2009). There was then a late, but rapid rise in primary care indicators: by week 49 (ending 12 December), rates of influenza-like illness (ILI) general practitioner (GP) consultation, as measured by the Royal College of General Practitioners (RCGP) Research and Surveillance Centre Weekly Returns Service had crossed their baseline value of 30/100,000 [3]. There continues to be uncertainty about the reasons for this unexpected upsurge in severe cases.

During the 2009 pandemic, the Health Protection Agency (HPA) and Department of Health (DH) in England ran a time-limited web-based hospital surveillance system for confirmed influenza cases in England (text S1) [4]. The scheme commenced in September 2009 and ceased operation in January 2010. In this paper, we present an analysis of data collected through the USISS sentinel network on hospital admissions with confirmed influenza in the 2010/11 season and compare the epidemiological picture to that seen during the 2009/10 pandemic.

## Methods

The USISS pilot scheme was established in October 2010 in order to determine the feasibility, cost and resources required to establish a routine hospital-based surveillance system for severe seasonal influenza, and establish the infrastructure in advance of any future pandemic. Voluntary enrolment of NHS Acute Trusts in England in the scheme commenced in December 2010. A case was defined as any person who was hospitalised and had a laboratory confirmed influenza A (H1 or H3) or B infection. Consultant microbiologists or infection control teams at each participating sentinel Trust submitted a weekly aggregate report of all cases admitted the previous week, broken down by influenza type, age group and maximum level of care. In order to maximise participation, enrolment in the scheme was not time-limited, with trusts able to provide retrospective data from 4 October (week 40) with their first data submission. A bi-weekly report was used to disseminate data collected by USISS to stakeholders. In addition, findings from USISS were shared on a regular basis with a wider audience of clinical specialists from across the UK.

The dataset analysed here comprises data from 4 October 2010 (week 40) to 13 February 2011 (week 6) and was compared to data from the pandemic period spanning 1 April 2009–6 January 2010. The median age of cases from each time period was compared using a Wilcoxon Rank Sum test. All statistical analysis was performed using Stata® (StataCorp Inc.) version 11.

Estimated influenza population hospitalisation rates were calculated from 19 Trusts who submitted data during both the 2009 pandemic and the 2010–2011 influenza season. To calculate the cumulative population age-specific hospitalisation rate the number of hospitalised confirmed cases in each age group admitted over each of these periods was divided by the total population of England in the same age group (as the hospital catchment populations were unknown). Although the time frames for each period of influenza circulation were not identical in length, they were taken to be equal for the purposes of this study, since a seasonal (or annual) cumulative rate of hospitalisation was being calculated. Population figures were the mid-year population estimate for England in 2009 obtained from the Office for National Statistics (ONS) [5]. The cumulative hospitalisation age-specific rate ratio was calculated using the 2009–2010 pandemic cumulative hospitalisation rate in each age group as the baseline. Rate ratio confidence intervals were calculated as described by Rothman KJ (1986) [6].

Data for the proportion of respiratory specimens submitted to HPA and NHS laboratories positive by PCR for influenza A/H1N1 (2009) by age group for the 2010–2011 influenza season (4 October 2010–14 February 2011) and pandemic period (20 April 2009–4 January 2010) were obtained from the HPA's DataMart system [7]. DataMart is a virological surveillance system that extracts influenza PCR results (positive and negative) from a network of 14 NHS and HPA laboratories across England. Chi^2^ tests were used to compare age-specific proportions of positive tests between the pandemic period and the 2010–2011 influenza season, as well as the proportion of cases admitted to critical care during both time periods.

Ethical approval was not sought for this scheme as it is part of routine national surveillance carried out under the NHS Act 2006 (section 251), which provides statutory support for disclosure of such data by the National Health Service, and their processing by the HPA, for the purposes of communicable disease control.

## Results

A sentinel network of 23 of 168 (13.7%) Acute Trusts from across England agreed to participate and submitted data to USISS for the study period. Eight of the ten regions of England were represented (North East England and the West Midlands were the two regions not represented). Participating Trusts varied in size, ranging from 316–1646 in-patient beds. All participating Trusts had intensive care units and paediatric services.

**Figure 1:**
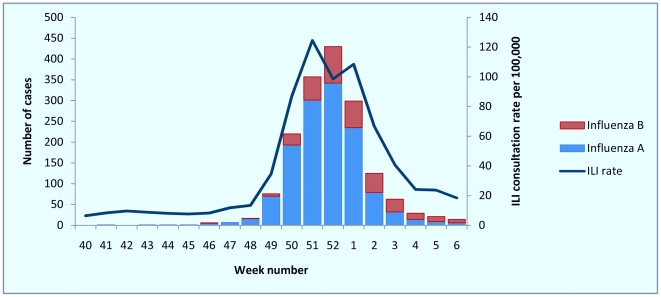
Number of hospitalised cases of influenza by type and by week of admission with weekly Royal College of General Practitioners (RCGP) influenza-like illness (ILI) consultation rate per 100 000 population: week 40 2010–week 6 2011, USISS sentinel network (n = 23), England.

Over the period from 4 October 2010 (week 40) to 13 February 2011 (week 6), a total of 1668 hospitalised cases of laboratory confirmed influenza were reported by participating Trusts. Admissions of cases, first reported in week 46 of 2010, started to increase in week 48 of 2010, peaking in week 52 of 2010 (figure 1). Case numbers declined steadily from week 1 of 2011 onwards, reaching a plateau by week 6 2011. The timing of hospitalisation of cases by week of admission resembled the rates of GP consultation for ILI. Community ILI consultation rate, however, increased starting in week 48 and peaked in week 51 (figure 1), one week earlier than admissions of hospitalised cases. ILI GP consultation rates then decreased and reached near-baseline levels (<30/100 000) by week 4.

Of the 1668 reported cases, 1260 (75.5%) were due to influenza A/H1N1 (2009), four (0.2%) were influenza A/H3N2, 49 (2.9%) were influenza A/unknown subtype and 355 (21.3%) were influenza B. The overall proportion of influenza B cases increased over the season (figure 1). During week 52, at the peak of activity, only 52 of 317 (16.4%) cases were influenza B; by week 3, however, influenza B comprised 31 of 63 (49.2%) reported cases. From week 3 to week 6, 66 of 127 (52.0%) reported cases were influenza B.

**Figure 2:**
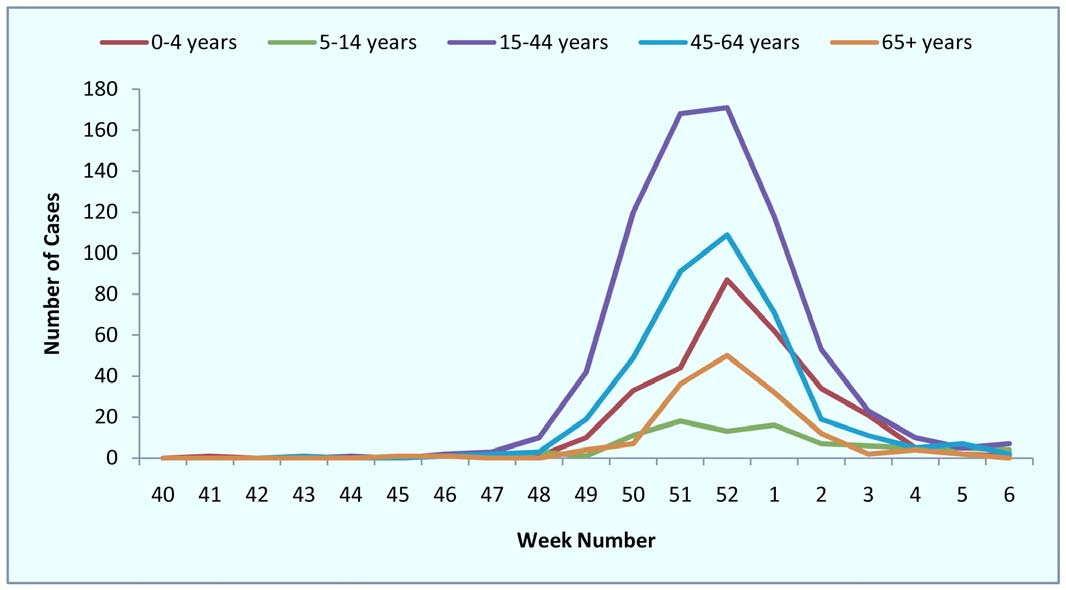
Number of hospitalised cases by age group and by week of admission: week 40 2010–week 6 2011, USISS sentinel network (n = 23), England.

Hospitalised cases occurred mainly in those under 65 years of age. Amongst the 1668 cases, 304 (18.2%) were age 0–4 years, 90 (5.4%) were age 5–14 years, 733 (43.9%) were age 15–44 years, 390 (23.4%) were age 45–64 years and 151 (9.1%) were aged over 64 years (figure 2). The median age of hospitalised influenza A/H1N1 (2009) cases at sentinel Trusts was 35 years (interquartile range (IQR) 18–52). In contrast, during the pandemic period, the median age of cases was 20 years (IQR 6–38) (p<0.0001). The median age of influenza B cases was 26 years (IQR 17–44), with their age distribution shown in table 1.

**Table 1:** Influenza hospitalisations and hospitalisation rates during the 2009 pandemic and during the 2010–11 influenza season, and influenza A/H1N1 (2009) rate ratio of hospitalisation during the 2010–2011 influenza season compared to the 2009 pandemic, USISS sentinel network (n = 19).

	2009 Pandemic (1 April 2009–6 January 2010)		2010/11 influenza season (4 October 2010–13 February 2011)				
Age Group	influenza A/H1N1 (2009) hospital cases	Cumulative hospitalisation rate/100,000 population	Influenza A/H1N1 hospital cases	Cumulative Hospitalisation rate/100,000 population	Influenza A/H1N1 (2009) rate ratio of hospitalisation (95% CI)	Influenza B hospital cases	Influenza B cumulative hospitalisation rate/100,000 population
0–4	82	2.6	169	5.3	2.1 (1.6–2.7)	63	2.0
5–14	101	1.7	36	0.6	0.4 (0.2–0.5)	41	0.7
15–44	162	0.8	496	2.3	3.1 (2.6–3.7)	117	0.6
45–64	53	0.4	275	2.1	5.2 (3.9–7.0)	44	0.3
65+	11	0.1	95	1.1	8.6 (4.6–16.1)	29	0.3
Total	409	0.8	1071	2.1	2.6 (2.3–2.9)	294	0.6

Based on data submissions from participating Trusts, the crude overall cumulative hospitalisation rate at all sentinel Trusts (n = 23) for influenza of all types for the period of week 40 2010–week 6 2011 was 3.2/100,000 general population. The hospitalisation rate decreased with age, from 9.5/100,000 population for cases 0–4 years of age to 1.8/100,000 population for cases over 64 years.

In order to compare influenza hospitalisations during the 2010/2011 season to hospitalisations during the 2009 pandemic, data from 19 Trusts that participated in these severe disease surveillance initiatives during both time periods was compared. At these Trusts 1071 influenza A/H1N1 (2009) hospitalised cases were reported during the 2010/11 season, compared to only 409 cases during the two 2009/10 pandemic waves (table 1). Both during the pandemic and during the 2010/11 season, reports were submitted both prospectively and retrospectively. Hospitalisation rates were calculated from 19 Acute Trusts that submitted data during both time periods. During the 2009 pandemic, the crude overall rate of hospitalisation at these Trusts for influenza A/H1N1 (2009), from 1 April 2009 to 6 January 2010, was 0.8/100,000 population (table 1). During the 2010/2011 season, from 4 October 2010 to 13 February 2011, the crude overall rate of hospitalisation for influenza A/H1N1 (2009) at the same Trusts was 2.1/100,000 population. As in the pandemic, there was a trend of decreasing hospitalisation rates with age, from 5.3/100,000 population for cases 0–4 years to 1.1/100,000 population for cases over 64 years. This compared to hospitalisation rates of 2.6/100,000 population for cases 0–4 years and 0.1/100,000 population for cases over 64 years during the 2009 pandemic. Surprisingly, in 2010/2011, the lowest hospitalisation rate was 0.6/100,000 population in cases aged 5–14 years, a marked difference from the pandemic period, when the hospitalisation rate for this age group was the second highest, at 1.7/100,000 population.

Overall, all age groups were 2.6 times more likely to be hospitalised with influenza A/H1N1 (2009) infection during the 2010/11 season than during the 2009 pandemic (confidence interval 2.3 to 2.9). The rate ratio for hospitalisation increased with age. The exception was those aged 5–14, who were significantly less likely to be hospitalised than during the 2009 pandemic. The decrease in hospitalisation rates in cases 5–14 years during the 2010/11 season was corroborated by laboratory data which showed that the proportion of positive influenza A/H1N1 (2009) specimens for the 5–14 year age group decreased from 43.3% during the pandemic to 17.6% in 2010/11 (p<0.0001) (table 2).

**Table 2:** Age-specific laboratory positivity rates for influenza during the 2009 pandemic and the 2010–2011 influenza season reported by the DataMart laboratory surveillance system, England.

	Influenza H1N1 (2009) proportion positive					Influenza B proportion positive	
Age group	2009 Pandemic		2010–2011			2010–2011	
	Total tests	Positive tests (%)	Total tests	Positive tests (%)	P-value	Total tests	Positive tests (%)
0–4 years	22616	2626 (11.6)	10979	1439 (13.1)	<0.0001	8495	459 (5.4)
5–14 years	14712	6376 (43.3)	2749	484 (17.6)	<0.0001	2192	535 (24.4)
15–44 years	35138	8506 (24.2)	13422	3968 (29.6)	<0.0001	9527	1167 (12.3)
≥45 years	22683	2459 (10.8)	15236	2874 (18.9)	<0.0001	10893	645 (5.9)
All ages	95149	19967 (21.0)	42386	8765 (20.7)	0.20	31107	2806 (9.0)

The crude overall rate of hospitalisation for influenza B from 4 October 2010 to 13 February 2011 in Acute Trusts that submitted data both during the pandemic and in 2010/11, was 0.6/100,000 population. As observed for influenza A/H1N1 (2009), the rate of hospitalisation decreased with age, from 2.0/100,000 population for cases 0–4 years to 0.3/100,000 population for cases over 64 years.

Of 1668 hospitalised influenza patients, 1431 (85.8%) were reported as admitted only to standard ward care (level 0, 1) and 237 (14.2%) to high dependency units ((HDU), level 2) or intensive care units ((ICU), level 3). Two hundred and six (86.9%) of the HDU/ICU admissions were influenza A/H1N1 (2009) cases, 11 were influenza A/unknown subtype and 20 were influenza B. The peak of HDU/ICU admissions in 2010/11 occurred in week 52, with 53 cases admitted comprising 22.4% of total HDU/ICU influenza admissions in 2010/11. HDU/ICU admissions occurred most frequently in 15–64 year olds, with 114 (48.1%) of 237 admissions in 15–44 year olds and 95 (40.1%) of 237 admissions 45–64 year olds.

## Discussion

The HPA successfully established a sentinel surveillance system for severe influenza, which demonstrated the impact and epidemiology of severe influenza infection in a timely manner in England in 2010/11. The system showed that during December 2010 to January 2011, there was a sharp rise in hospitalised cases of confirmed influenza, which mirrored other indicators of influenza activity. The majority of severe cases were due to influenza A/H1N1 (2009) infection, with a minority due to influenza B. As seen in previous years, the proportion of influenza B cases increased with time over the 2010/11 season. Rates of severe disease were higher than observed in the 2009/10 pandemic. The data indicates an increase with age in the risk of hospitalisation as well as a shift in the age-distribution of severe influenza A/H1N1 (2009) cases away from the 5–14 year age group, which had the highest attack rate during the 2009/10 pandemic, to older age groups during the 2010/11 seasonal influenza season. A significant number of influenza B cases occurred in 15–44 year olds; however the highest rates of hospitalisation for influenza B were in 0–4 year olds, followed by 5–14 year olds.

Although this study provides an estimate of the burden of severe influenza during the 2010/11 season, there are several limitations to these data. Firstly data analysis was limited by the lack of individual-level data on underlying risk factors for severe disease, course of illness, mortality outcome and antiviral use, restricting the ability to monitor epidemiological changes in the population vulnerable to severe disease and linkage with other records to assess the influence of vaccination status or clinical history. Secondly since aggregate data reporting is less time consuming than individual level data reporting, it is possible that the overall increase in cases observed in 2010/11 was partially due to more complete case reporting than during the 2009 pandemic, when individual-level data was reported. Thirdly, in contrast to during the pandemic, when 129 of 168 (77%) Trusts participated in surveillance (text S1) [4], USISS was developed as a sentinel scheme, with participation of 23 of 168 (13.7%) of Trusts. Hence the comparative analysis has been restricted to the same 19 trusts in both periods. As a result, although the hospitalisation rates reported here provide for meaningful comparisons of age-specific rates between different time periods, they are lower than the true hospitalisation rates since the denominator is the total population of England.

The findings are corroborated by other influenza activity indicators. These are helpful in addressing potential confounding due to changes in health care seeking behaviour or criteria for hospitalisation between the two time periods, which were difficult to measure directly. Community indicators, such as general practitioner influenza-like illness consultations rates, and syndromic surveillance indicators, such as telephone calls to the telephone help-line, NHS Direct, both indicated high levels of activity in 2010/11 [8], [9]. RCGP GP consultation rates continued to rise and reached a peak of 124.4/100,000 in week 51, the highest rate seen in 10 years apart from during the summer of the 2009 pandemic [8]. A similar trend was seen with various other syndromic surveillance indicators, with cold/flu calls to the telephone health advice service, NHS Direct, peaking in week 51 [8]. In addition to high levels of influenza in primary care and the community, there was also a sharp increase in severe influenza cases. By week 1, 783 critical care beds (22.5%) in England were occupied by suspected or confirmed influenza patients [10], with particular pressure on Extra Corporeal Membrane Oxygenation (ECMO) facilities across England. Although some widespread influenza activity with possible localised hotspots had been expected due to the H1N1 (2009) virus during the 2010–2011 influenza season, the abrupt increase in activity across the UK this season was surprising. Following the decline of influenza activity at the end of the second wave in 2010, serological studies undertaken in February 2010 had indicated that there was substantial immunity in the population to the pandemic H1N1 strain, particularly in younger age-groups [11]. The influenza A/H1N1 (2009) activity that began in December 2010 occurred despite virological analyses indicating that there was no significant antigenic change in the strain since 2009/10 [12]. Overall, however, there was an increase with age in the risk of being hospitalised compared to 2009, expressed as the risk per head of population, despite a considerable proportion of the population at risk of severe disease having been immunised against A/H1N1 (2009). As in 2009, H1N1 (2009) cases aged 0–4 years in 2010/11 were the most likely to be hospitalised. This is corroborated by other data, such as GP consultation rates and age-specific laboratory positivity rates; however it is possible that there was a behavioural component to this finding. Rates of hospitalisation were also elevated in the 15–64 year age-group, with those 64 years and older the least likely to be hospitalised. There was, however, a marked decrease in the influenza A/H1N1 (2009) hospitalisation rate for the 5–14 year age-group in 2010/11 compared with 2009/10. This was the age group with the highest age-specific positivity in the virological surveillance schemes in 2009/10 (table 2), while during 2010/11, the highest positivity observed in the 15–44 and over ≥45 year age-groups. Thus, despite being the group most affected during the pandemic, likely due to lack of prior immunity and increased risk of exposure, influenza A/H1N1 (2009) transmission seems to have shifted from the 5–14 age-group to older age-groups in 2010/11, as a result of an increase in immunity in children and young adolescents. It has previously been shown that the risk of influenza-related ICU admissions and fatalities increases with age [13]. Older age groups are more likely than children to develop severe influenza requiring hospitalisation and critical care due to a higher prevalence of underlying clinical risk factors, which may partly explain the observed increase in impact (text S1) [4]. Another potential contributory factor for the apparent increase in severity compared to the 2009/10 pandemic may have been an increase in bacterial co-infections in influenza A/H1N1 (2009) cases [14]. The proportion of H1N1 (2009) cases in HDU/ICU was significantly higher in 2010/11 than during the pandemic, when 253 of 2416 (10.5%) of cases were admitted into HDU or ICU (p = <0.0001) (text S1) [4].

A shift in the incidence of both uncomplicated and severe influenza to older age groups as well as an increase in severe influenza activity was also seen in several other European countries in 2010/11. In Greece, 2010/11 ILI rates were higher than in previous seasons (apart from the 2009 pandemic), with an increase in the median age of sentinel ILI cases compared to the 2009 pandemic. An increase in both influenza-related ICU admissions and fatalities was noted in 2010/11 compared to the 2009 pandemic, with older age groups affected in 2010/11 [15]. In Ireland, ILI rates in the 0–15 year age-group were significantly lower in 2010/11 than during the 2009 pandemic [16]. This phenomenon has been associated not only with the 2009 pandemic, but also with previous pandemics, such as those seen in 1918, 1957 and 1968 [17], [18].

This study shows evidence of an overall increase with age in the risk of hospitalisation compared to the 2009/10 pandemic period, as well as a shift in age distribution for severe influenza infection during the 2010–2011 influenza season. This demonstrates the benefit of hospital surveillance in rapidly determining which groups (e.g. age or with underlying health conditions) are at risk of developing severe disease and its potential for providing early warning of atypical patterns of severe acute respiratory illness presenting to hospital. Furthermore through weekly submissions from Acute Trusts, data obtained through the USISS pilot has the ability to provide a timely indication of changes in the epidemiology of severe influenza, and thus potentially contribute to the refinement of clinical care guidelines and support of policy.

## Supporting Information

Text S1 Hospitalization in two waves of pandemic influenza A(H1N1) in England.
(PDF)
