# Supplementary material for: A New Sentinel Surveillance System for Severe Influenza in England Shows a Shift in Age Distribution of Hospitalised Cases in the Post-Pandemic Period
Source: PLoS One. 2012 Jan 23;7(1):e30279. doi: 10.1371/journal.pone.0030279 (PMC3264602; doi:10.1371/journal.pone.0030279)
Supplement: Text S1 — Hospitalization in two waves of pandemic influenza A(H1N1) in England. (PDF) [file pone.0030279.s001.pdf]

Epidemiol. Infect., Page 1 of 10. © Cambridge University Press 2010
doi:10.1017/S0950268810002657

Hospitalization in two waves of pandemic influenza A(H1N1) in England

C. N. J. CAMPBELL[1*], O. T. MYTTON[2], E. M. McLEAN[1], P. D. RUTTER[2], R. G. PEBODY[1], N. SACHEDINA[2], P. J. WHITE[1,4], C. HAWKINS[3], B. EVANS[1], P. A. WAIGHT[1], J. ELLIS[1], A. BERMINGHAM[1], L. J. DONALDSON[2] AND M. CATCHPOLE[1]

[1] *Health Protection Agency, Centre for Infections, London, UK*
[2] *Department of Health, Richmond House, London, UK*
[3] *Health Protection Agency, Local and Regional Services, UK*
[4] *MRC Centre for Outbreak Analysis & Modelling, Department of Infectious Disease Epidemiology, Imperial College London, UK*

(*Accepted 26 October 2010*)

SUMMARY

Uncertainties exist regarding the population risks of hospitalization due to pandemic influenza A(H1N1). Understanding these risks is important for patients, clinicians and policy makers. This study aimed to clarify these uncertainties. A national surveillance system was established for patients hospitalized with laboratory-confirmed pandemic influenza A(H1N1) in England. Information was captured on demographics, pre-existing conditions, treatment and outcomes. The relative risks of hospitalization associated with pre-existing conditions were estimated by combining the captured data with population prevalence estimates. A total of 2416 hospitalizations were reported up to 6 January 2010. Within the population, 4·7 people/100 000 were hospitalized with pandemic influenza A(H1N1). The estimated hospitalization rate of cases showed a U-shaped distribution with age. Chronic kidney disease, chronic neurological disease, chronic respiratory disease and immunosuppression were each associated with a 10- to 20-fold increased risk of hospitalization. Patients who received antiviral medication within 48 h of symptom onset were less likely to be admitted to critical care than those who received them after this time (adjusted odds ratio 0·64, 95% confidence interval 0·44–0·94, $P=0{\cdot}024$). In England the risk of hospitalization with pandemic influenza A(H1N1) has been concentrated in the young and those with pre-existing conditions. By quantifying these risks, this study will prove useful in planning for the next winter in the northern and southern hemispheres, and for future pandemics.

Key words: Infectious disease epidemiology, influenza, influenza A, pandemic, surveillance.

INTRODUCTION

The emergence of the first pandemic of influenza for 40 years proved less severe than had been anticipated in many governments' plans [1, 2]. As a result, most people [3] who were infected with pandemic influenza A(H1N1) suffered a short, self-limiting illness with no complications [4]. A proportion, however, suffered a serious illness resulting in hospitalization [5, 6]. For some this involved time in a critical care facility [6–10].

* Author for correspondence: Dr C. N. J. Campbell, HPA Centre for Infections, 61 Colindale Avenue, London NW9 5EQ, UK. (Email: Col.campbell@rocketmail.com)

Early clinical reports and, later, analysis of aggregated data from around the world in the first phase of the new pandemic, provided insight into the nature of the complications that were arising particularly in children and younger adults [10–14]. This age profile for severe disease was different to seasonal influenza, which affects those aged ⩾65 years disproportionately [15]. Key risk factors (such as pre-existing medical conditions, younger age and pregnancy) for hospitalization following infection with pandemic influenza A(H1N1), were reported for Mexico, North America and Australasia [5, 7, 8, 16].

Uncertainties remain about the risks for hospitalization at the population level and for people with particular pre-existing conditions. Information on these risks is essential for assessing the adequacy of health service capacity (particularly of critical care) as well as clinical and public health interventions. The pandemic influenza A(H1N1) virus is expected to return in the Northern and Southern hemisphere 2010 influenza seasons [17].

We have gathered and analysed data on patients hospitalized with pandemic influenza A(H1N1) within a whole country during the main period of disease activity with the aim of clarifying some of these key uncertainties.

METHODS

A surveillance system for all hospital in-patients with pandemic influenza A(H1N1) in England was established in September 2009. It was designed to identify and quantify the risk factors for severe illness and to detect trends in virus behaviour. Hospitals already participating in a research project on pandemic influenza A(H1N1) were not invited to contribute to this surveillance system, to minimize the reporting burden on clinicians.

Consultant microbiologists in each hospital were asked to submit a standardized dataset for any case of pandemic influenza A(H1N1) admitted to their hospital. A case was defined as any person formally admitted to hospital (regardless of duration of stay) who had laboratory confirmation [by polymerase chain reaction (PCR) testing] of pandemic influenza A(H1N1) during or prior to their hospital admission.

The dataset included: demographic information (date of birth, sex), pre-existing medical conditions (by organ system, pregnancy and immunosuppression), treatment (antiviral medication use), dates of admission and discharge (to hospital and to critical care if relevant), and complications, as well as patient identifiers [National Health Service (NHS) number, hospital number, name]. Missing data were excluded from the denominator where appropriate.

Data gathering was via a secure web-based portal, which prompts clinicians for missing data fields. Incomplete records were followed up by telephone or by linking with other laboratory and field data. Additional cases were identified through the Health Protection Agency's (HPA) regional microbiology network.

Explicit ethical approval was not sought as this data collection was part of routine pandemic surveillance. Surveillance was carried out under the NHS Act 2006 (section 251), which provides statutory support for disclosure of such data by the NHS, and their processing by the HPA, for communicable disease control.

Prevalence of pre-existing medical conditions

The population prevalence of specific pre-existing medical conditions (excluding pregnancy) was estimated from information provided by English general practitioners (GPs) to the Department of Health-HPA (DoH-HPA) influenza vaccine uptake monitoring system [18]. For the population aged 6 months to 64 years, a breakdown by individual pre-existing condition is available (based on data provided by 96·2% of all English GP practices) [3]. For the population aged ⩾65 years, the number of people with a pre-existing medical condition was extrapolated from data provided by 79·4% of GP practices (provisional data provided by the DoH). A breakdown by pre-existing condition was not available for people aged ⩾65 years.

The point prevalence of pregnant women was estimated using the sum of the published number of maternities (births and stillbirths) and an estimate of the number of miscarriages and abortions each year [19]. An estimate of the female population of childbearing age (15–44 years) was used to estimate the annual number of miscarriages or abortions, assuming a 4% abortion/miscarriage rate [20]. To calculate the point prevalence of pregnant women, 9/12 of the annual number of maternities (assuming a pregnancy of 9 months' duration) was added to 3/12 of the annual number of miscarriages or abortions (assuming a mean duration of 3 months). The number of pregnancies in each trimester was calculated, assuming that maternities were divided equally between

trimesters and that all miscarriages and abortions occurred in the first trimester.

Estimated hospitalization rates for cases of pandemic influenza A(H1N1)

Estimated hospitalization rates for cases of symptomatic pandemic influenza A(H1N1) within the population were calculated for the period 1 April 2009 to 6 January 2010. A 1-week lag period was assumed from disease onset to hospital admission.

Throughout the pandemic, the HPA provided estimates of the total number of symptomatic cases of pandemic influenza A(H1N1). This cumulative estimate was used as the denominator to calculate the estimated hospitalization rates for cases with pandemic influenza A(H1N1). The method of estimating the number of symptomatic cases incorporates the number of people consulting their GP with influenza-like illness, the number using a national internet and telephone-based system to obtain antiviral medication (the National Pandemic Flu Service), the proportion of each of these groups with laboratory-confirmed pandemic influenza A(H1N1) in a tested sample (the positivity rate) and an estimate of the proportion of those with symptomatic illness in the population who do not seek medical attention via either of these routes [21]. Positivity and consultation rates were stratified by age, providing case estimates by age group. Upper and lower estimates around the central estimate reflect the uncertainty inherent in estimating case numbers.

Estimated hospitalization rates for cases of pandemic influenza A(H1N1) were calculated using the central estimate of cases as the denominator. The upper and lower estimates of cases were used to calculate lower and upper estimates, respectively, of the estimated hospitalization rate for cases. A 95% confidence interval (CI) was calculated around these estimates to account for the uncertainty around the observed number of hospitalizations. The ranges presented in this paper refer to the upper 95% confidence limit of the upper estimated rate and the lower 95% confidence limit of the lower estimated rate.

In addition to the calculating the estimated hospitalization rates for cases of pandemic influenza A(H1N1), estimated hospitalization rates for the population were calculated. The estimated hospitalization rate for the population was estimated by dividing the number of hospital admissions due to pandemic influenza A(H1N1) by the population. Population estimates were taken from the Office for National Statistics for 2007 [20].

To estimate the number of clinical cases with different pre-existing conditions, it was assumed that the risk of acquiring pandemic influenza A(H1N1) was the same for the general population and for those with pre-existing medical conditions. It was assumed that infants aged <6 months represent half the cases aged <1 year.

Relative risk

Pooled Mantel–Haenszel age-adjusted relative risks were calculated for each pre-existing condition with the exception of pregnancy. The denominator data were available in the age groups 6 months to <16 years and 16–64 years. Consequently, children have been defined as being aged <16 years. The relative risk for pregnancy was calculated by comparing the hospitalization rate for pregnant women with the rate for women of childbearing age. In all cases the comparison group was those with no risk factors. The attributable fraction in the exposed and the population attributable fraction were estimated for each pre-existing condition.

RESULTS

In total, 2416 hospitalized cases were reported from 1 April 2009 to 6 January 2010. Reports were made by 77% of eligible hospital trusts (129/168). Many of the trusts which did not submit data were already reporting to a separate research project. Those trusts that did not make reports were no different from those that did in complexity of referrals (secondary *vs.* tertiary facility) or number of beds (mean 619 *vs.* 711, $P=0{\cdot}2$).

The crude overall rate of hospitalizations for the 8-month period encompassing the two waves of the pandemic was 4·6/100 000 population (Table 1). The rate of hospitalization decreased markedly with increasing age. The median age of admitted patients was 20 years [interquartile range (IQR) 6-38] and 48% (1160/2411) were male. The median length of hospital stay was 2 days (IQR 1-5). Length of stay was greater for adults (median 3 days, IQR 1-6) than for children (median 1 day, IQR 1-3), $P<0{\cdot}001$.

Estimated cases and hospitalizations occurred in two waves (Fig. 1). The first wave peaked in mid-July 2009, the second in late October 2009. The greatest number of admissions within the ten English NHS

Table 1. *Laboratory-confirmed hospitalization and critical care admission rates for pandemic influenza A(H1N1) by age group*

Age group (years)	Hospitalized cases	Hospitalization rate/100 000 population (95% CI)	Critical care admissions (percent of hospitalized patients)	Critical care admission rate/100 000 population (95% CI)
<1	184	27·6 (24·0–32·2)	5 (2·7%)	0·7 (0·4–2·2)
1–4	305	12·4 (11·0–13·9)	5 (1·6%)	0·2 (0·1–0·5)
5–14	476	8·1 (7·5–8·9)	31 (6·5%)	0·5 (0·4–0·7)
15–24	408	5·9 (5·4–6·5)	35 (8·6%)	0·5 (0·4–0·7)
25–44	558	3·9 (3·6–4·3)	73 (13·1%)	0·5 (0·4–0·6)
45–64	351	2·7 (2·5–3·0)	77 (21·9%)	0·6 (0·5–0·8)
⩾65	98	1·2 (1·0–1·5)	18 (18·4%)	0·2 (0·1–0·5)
All ages	2380	4·6 (4·4–4·9)	244 (10·3%)	0·5 (0·4–0·6)

CI, Confidence interval.

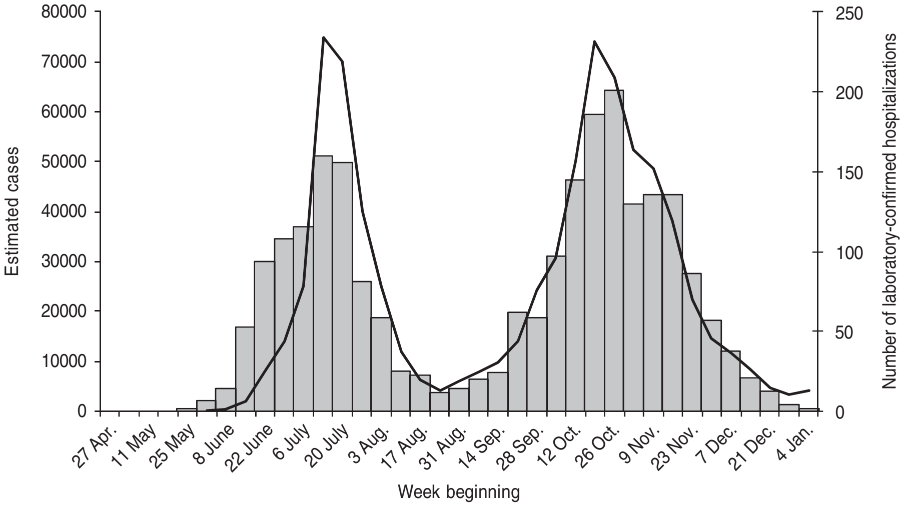

Fig. 1. Estimated cases (——) of pandemic influenza A(H1N1) within the population and number of laboratory-confirmed hospitalizations (▢) by week.

regions was reported from hospitals in the West Midlands (477, 8·8 cases/100 000 population) and in London (401, 5·3 cases/100 000 population).

The estimated hospitalization rate for cases of pandemic influenza A(H1N1) fell over the course of the pandemic. In June 2009 it was 1580/100 000 cases and by October 2009 it had fallen to 260/100 000 cases (test for trend $P<0{\cdot}0001$). Overall, 310 (range 120–680) of every 100 000 estimated cases were admitted to hospital. Those at the extremes of age had the highest rates of hospitalization (Fig. 2).

Risk factors for hospitalization

Information on pre-existing medical conditions was available for 91% (2209/2416) of hospitalized patients. Of these 58% (1296/2209) had one or more pre-existing conditions. This proportion increased with age (Fig. 3*a*). Patients with pre-existing conditions had a greater length of stay (median 3 days, IQR 1-6) than those without (median 1 day, IQR 1-3) ($P<0{\cdot}001$).

The relative risk of hospitalization was ten times greater for those with a pre-existing medical condition compared to those without, in those aged 6 months to 64 years (Table 2). The pre-existing conditions conferring the highest relative risks of hospitalization in those aged 6 months to 64 years were immunosuppression, chronic renal disease, chronic neurological disease and chronic respiratory disease (Table 2).

Twenty-one percent of all women aged 15–44 years were pregnant at the time of admission. Pregnancy

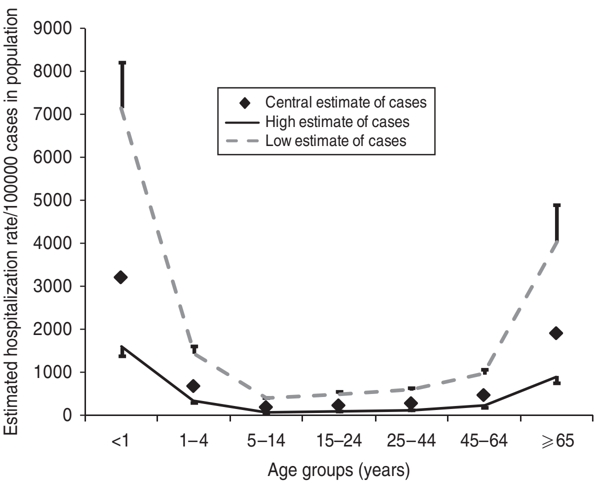

Fig. 2. Estimated hospitalization rates for cases of pandemic influenza A(H1N1) in different age groups within the population.

conferred a 7·8-fold greater risk of admission than that for all females of childbearing age (Table 3). The risk of admission was greater during the second and third trimesters than the first trimester. The majority of pregnant women had no other pre-existing conditions (72%, 97/135). The most common pre-existing conditions in pregnancy were asthma (23/135), immunosuppression (5/125) and diabetes (4/133).

Critical care admission

Overall, 33 of every 100 000 estimated cases within the population were admitted to critical care. Of the patients admitted to hospital, 10·5% were admitted to critical care. The proportion of hospital patients admitted to critical care increased with age (Table 1). The median length of stay in critical care was 5 days (IQR 2–12 days, $n=136$). The length of stay in critical care was not affected by the presence of pre-existing conditions (median 5 days *vs.* 4·5 days for no pre-existing condition, $P=0{\cdot}55$).

Pre-existing conditions were significantly more common in patients admitted to critical care than in hospitalized cases as a whole (79% *vs.* 54%, $P<0{\cdot}001$; Fig. 3). Of patients admitted to critical care, the proportion with a pre-existing condition was similar across the age groups for those aged <65 years (test for trend $P=0{\cdot}59$; Fig. 3*b*). A lower proportion of those aged ⩾65 years had a pre-existing conditions than those aged ⩽64 years (60% *vs.* 82%, $P=0{\cdot}01$). By contrast, for hospitalized patients as a whole, the proportion with a pre-existing condition increased with age (Fig. 3*a*).

Use of antiviral medication

Data on antiviral medication was available for 81% (1959/2416) of cases. Antiviral medication was administered during the hospital stay for 67% (1299/1927) of patients. Antiviral medication had been started within the recommended 48-h window after symptom onset in 44% (617/1416) and prior to admission in 12% (213/1826) of cases. Patients who received antiviral medication within the recommended 48 h after onset of symptoms were less likely to be admitted to critical care than those who received them after 48 h, after adjusting for age, sex and underlying risk factors [odds ratio (OR) 0·68, 95% CI 0·47–0·99, $P=0{\cdot}047$]. No effect on mortality was observed (OR 0·77, 95% CI 0·42–1·43, $P=0{\cdot}41$).

Complications

Complications were reported in 349 cases. The most commonly reported complications were pneumonia (321/2416, 13·3%), acute respiratory distress syndrome (44/2416, 1·8%), renal failure (31/2416, 1·3%), shock (25/2416, 1·0%) and encephalopathy (10/2416 0·4%).

Information on the conclusion of the hospital admission was available in 93% of cases (2242/2416). Of these, 79 deaths were reported. This gives a hospital case-fatality rate of 3·5%. The hospital case-fatality rate was highest in those aged >64 years (20%) and lowest in those aged <5 years (0·4%). The hospital case-fatality rate was significantly higher for those with pre-existing conditions compared to those without (5·1% *vs.* 1·4%, $P<0{\cdot}001$).

DISCUSSION

Over two waves, pandemic influenza A(H1N1) in England caused 4·7/100 000 people to be hospitalized. This rate is lower than that reported in other countries, including Australia (22·8/100 000 population), The Netherlands (13·1) and Argentina (27·5) [16, 22]. England has taken an aggressive approach to the pandemic, including a media campaign promoting hand-washing and early widespread access to antiviral medication for cases within the community. This may have contributed to the low hospitalization rate. Alternatively, the lower rate of hospitalization in England may be explained by international differences in case definition, reporting systems or thresholds for admission.

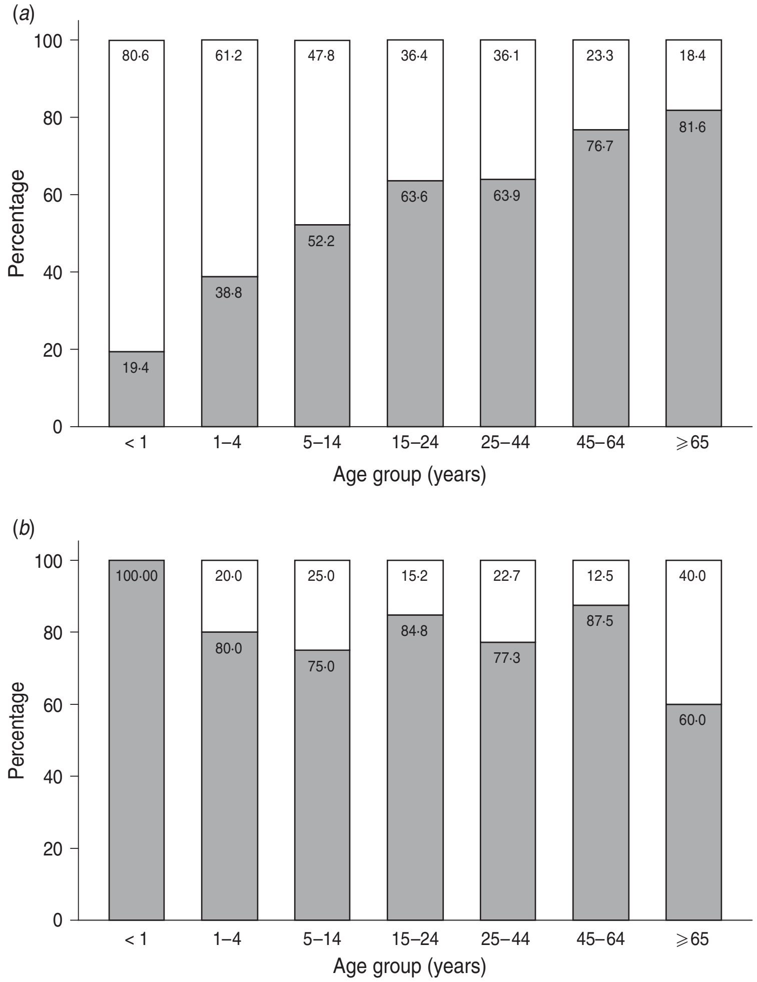

Fig. 3. Distribution of pre-existing conditions by age group for (*a*) all pandemic influenza A(H1N1) hospitalizations and (*b*) critical care admissions. ■, With underlying condition; □, without underlying condition.

We have calculated the relative risk of hospitalization associated with different pre-existing conditions. Chronic kidney disease, chronic neurological disease, chronic respiratory disease and immunosuppression are associated with a 10- to 20-fold increased risk of hospitalization. Reports of pregnancy as a risk factor for pandemic influenza were also substantiated by this study with the greatest risk for those in the third trimester. Other studies have identified chronic respiratory disease and chronic neurological disease as the most common pre-existing conditions in those hospitalized or dying [23–25]. These studies have not quantified the risk of hospitalization for people with these conditions. By contrast, our study takes account of chronic disease prevalence in the population. In so doing, we show that other, less common, pre-existing conditions, such as chronic kidney disease and immunosuppression, are associated with a similar, or even greater, risk of hospitalization than chronic respiratory disease and chronic neurological disease. It is possible that different admission criteria applied to those with pre-existing conditions could contribute to the higher hospitalization rates. However, the high risk of severe disease in this group suggests this contribution is likely to be small. Quantifying these risks is important. It can guide advice to the public, particularly for those with existing illnesses. Quantifying the risks can also help clinicians in decisions about the timing of treatment of high-risk patients. It can also guide policy decisions about vaccination.

A small number of studies have estimated the total number of cases of pandemic influenza A(H1N1) within the population so that it could be used as a denominator for calculating hospitalization rates for affected people. Our study found that an estimated 0·31% of cases of pandemic influenza A(H1N1) were hospitalized. This is similar to New Zealand (0·30%) and the USA (0·44%) [16, 26]. Our age-specific

Table 2. *Hospitalization rates, relative risk and attributable fractions for different pre-existing conditions*

	Population (1000s)	Estimated cases (1000s)	Hospital admissions	Hospitalization rate/100 000 population (95% CI)	Relative risk (95% CI)	Estimated hospitalization rate/100 000 cases within the population (range)	Attributable fraction among exposed (%)	Population attributable fraction (%)
**6 months to 64 years**								
No pre-existing conditions	39 370	683·8	1005	2·6 (2·4–2·7)	1·0	147·0 (66·1–329·2)		
Any Pre-existing conditions	4579	79·5	1016	22·2 (20·8–23·6)	10·3 (9·4–11·3)	1277·4 (574·6–2858·4)	88·5	44·5
Chronic kidney disease	182	3·2	58	31·7 (24·1–41·0)	17·5 (13·4–22·9)	1826·8 (664·6–4943·9)	85·9	2·5
Chronic heart disease	688	11·9	85	12·4 (9·9–15·3)	6·1 (4·8–7·6)	711·6 (272·2–1849·7)	63·8	2·7
Chronic respiratory disease	2015	35·0	593	29·4 (27·1–31·9)	12·1 (10·9–13·4)	1693·6 (747·1–3859·9)	88·4	25·9
Chronic liver disease	139	2·4	22	15·8 (9·9–23·9)	8·9 (5·8–13·5)	908·3 (272·7–2882·1)	71·1	0·8
Diabetes	1010	17·6	75	7·4 (5·8–9·3)	4·2 (3·3–5·3)	427·4 (161·0–1126·9)	38·9	1·4
Immunosuppression	373	6·5	132	35·4 (29·6–41·9)	18·4 (15·3–22·1)	2035·8 (816·1–5063·5)	87·7	5·7
Chronic neurological disease	430	7·5	120	27·8 (23·1–33·3)	14·3 (11·8–17·2)	1603·2 (636·8–4024·0)	84·3	5·0
Total	43 949	763·3	2217	5·0 (4·8–5·3)		290·4 (133·3–637·6)		
**⩾65 years**								
No pre-existing conditions	4051	2·6	22	0·5 (0·3–0·8)	1·0	853·0 (256·3–2710·9)		
Any pre-existing conditions	4234	2·7	65	1·5 (1·2–2·0)	2·8 (1·7–4·6)	2411·0 (892·9–6437·0)	64·6	48·3
Total	8285	5·3	101	1·2 (1·0–1·5)		1914·7 (748·0–4885·5)		

CI, Confidence interval.

Population estimates are mid-2008 estimates [18]. Cases may have more than one pre-existing condition. The relative risk is calculated relative to the group with no pre-existing conditions for that age group. For the age group 6 months to 64 years a pooled Mantel–Haenszel age-adjusted relative risk was calculated.

Table 3. *Hospitalization rates and relative risk for pregnant women* (*aged 15–44 years*)

Women (aged 15–44 years)	Population (1000s)	Estimated cases (1000s)	Hospital admissions	Hospitalization rate/100 000 population (95% CI)	Relative risk (95% CI)	Estimated hospitalization rate/100 000 cases within the population (range)
No pre-existing conditions	9449	168·9	286	3·0 (2·7–3·4)	1·0	1·7 (0·7–4·0)
Pregnant women	600	11·0	141	23·0 (19·4–27·1)	7·8 (6·4–9·6)	12·9 (5·2–31·8)
First trimester	270	4·9	13	4·7 (2·5–8·1)	1·0	2·6 (0·7–94·8)
Second trimester	165	3·0	25	14·8 (9·6–21·8)	3·1 (1·6–6·1)	8·3 (2·6–25·5)
Third trimester	165	3·0	58	34·3 (26·1–44·3)	7·2 (4·0–13·2)	19·2 (7·0–51·7)

CI, Confidence interval.
The number of women of childbearing age (15–44 years) with no pre-existing conditions is an estimate based on the proportion of the whole population aged 6 months to 64 years who do not have any pre-existing conditions.

estimated hospitalization rates for cases of pandemic influenza A(H1N1) show a U-shaped distribution by age. Both the elderly and the young have high rates of admission when infected with pandemic influenza. This distribution is similar to that observed for deaths in the current and previous pandemics [1, 24, 27]. In contrast, the hospitalization rate within the overall population declines as age increases. This reflects the very low clinical incidence of infection in older people, probably due to pre-existing immunity [28].

Overall, 10·5% of hospitalized cases were admitted to critical care, with the proportion requiring critical care increasing with age. Those who received antiviral medication within the recommended 48-h window after symptom onset were less likely to be admitted to critical care than those receiving antiviral medication after this window. It is of concern, therefore, that early antiviral use in this study was low. Use of antiviral medication at any point during the hospital stay (67%) was lower than observed in France (81%) and the USA (75%) [5, 29]. This may reflect lack of familiarity with antiviral medication, a perception of low efficacy among clinicians, or a generally negative attitude fuelled by media coverage of 'side-effects'. Alternatively, it may reflect poor documentation in clinical notes.

England had a targeted vaccination campaign. In the first phase, vaccination was offered to those with pre-existing medical conditions including pregnancy. In the second phase, vaccination was offered to all children aged <5 years. This paper supports this prioritization. Those with pre-existing medical conditions had the highest rates of hospital admission. By age group, hospitalization rates were highest for those aged <5 years.

Our study has a number of strengths. We have collected data on a whole country, capturing key epidemiological characteristics for a large number of hospital admissions. By using an estimate of the prevalence in the community, we have been able to quantify the relative risks associated with pre-existing conditions. As with similar surveillance systems operating during a pandemic, under-ascertainment of cases may have occurred. This is likely to have occurred equally across age and pre-existing conditions. While the absolute rate of hospitalization may be an under-estimate, the relative risk estimates are likely to be valid. The case estimates used to calculate the estimated hospitalization rates for cases of pandemic influenza A(H1N1) are uncertain. This is reflected in the large confidence intervals.

Setting up and maintaining a national surveillance system for hospitalizations due to pandemic influenza A(H1N1) requires high level political support. By using a secure electronic portal for reporting an additional burden was placed on clinicians in acute hospitals who were busy responding to the pandemic. In including so many hospitals it was difficult to quality-assure the system. While the system was able to capture key data, a sentinel hospital surveillance network is being piloted to capture data for the forthcoming influenza season.

CONCLUSIONS

In England the risk of hospitalization with pandemic influenza A(H1N1) has been concentrated in the young and those with pre-existing conditions. Establishing a national hospital surveillance system has allowed us to quantify the risk factors for

hospitalization. This is valuable for patients, clinicians and policy makers, informing decision-making. This information will be essential when planning for the next winter, both in the southern and northern hemispheres, and for future pandemics.

ACKNOWLEDGEMENTS

We are grateful to the many clinicians in England who supplied clinical information on their patients and to Esther Adeyemi for coordinating contacts with hospital clinicians. We are also indebted to colleagues at the HPA Centre for Infections; Liz Miller, Nick Phin, John Watson, Bev Paterson, Maria Zambon, Pia Hardelid and the CfI Statistics team; Christine McCartney and colleagues at the Regional Microbiology Network for invaluable contributions; Sam Bracebridge and colleagues in HPA Local and Regional Services; Asaf Niaz and the development team for building the data collection tool.

DECLARATION OF INTEREST

All authors have support from their respective institutions for the submitted work. L. J. D., as Chief Medical Officer advised government on public health policy, P. J. W. is a member of UK Government's Scientific Advisory Group for Emergencies (SAGE) and Scientific Pandemic Influenza Advisory Committee modelling sub-group (SPI-M); O. J. M., P. D. R., and N. S. advised the Chief Medical Officer.

REFERENCES

1. **Donaldson LJ, *et al.*** Mortality from pandemic A/H1N1 2009 influenza in England: public health surveillance study. *British Medical Journal* 2009; **339**: b5213.
2. **Bishop JF, Murnane MP, Owen R.** Australia's winter with the 2009 pandemic influenza A (H1N1) virus. *New England Journal of Medicine* 2009; **361**: 2591–2594.
3. **Pebody RG, *et al.*** Influenza vaccination coverage in England, 2000–2008. *Eurosurveillance* 2008; **13**(51): pii = 19074.
4. **WHO.** (http://www.who.int/wer/2009/wer8421.pdf). *WHO Weekly Epidemiological Record* 2009; **84**: 185–196.
5. **Jain S, *et al.*** Hospitalized patients with 2009 H1N1 influenza in the United States, April–June 2009. *New England Journal of Medicine* 2009; **361**: 1935–1944.
6. **CDC.** Intensive-care patients with severe novel influenza A (H1N1) virus infection – Michigan, June 2009. *Morbidity and Mortality Weekly Report* 2009; **58**: 749–752.
7. **Webb SA, *et al.*** Critical care services and 2009 H1N1 influenza in Australia and New Zealand. *New England Journal of Medicine* 2009; **361**: 1925–1934.
8. **Dominguez-Cherit G, *et al.*** Critically Ill patients with 2009 influenza A(H1N1) in Mexico. *Journal of the American Medical Association* 2009; **302**: 1880–1887.
9. **Davies A, *et al.*** Extracorporeal membrane oxygenation for 2009 influenza A(H1N1) acute respiratory distress syndrome. *Journal of the American Medical Association* 2009; **302**: 1888–1895.
10. **Perez-Padilla R, *et al.*** Pneumonia and respiratory failure from swine-origin influenza A (H1N1) in Mexico. *New England Journal of Medicine* 2009; **361**: 680–689.
11. **CDC.** Hospitalized patients with novel influenza A (H1N1) virus infection – California, April–May, 2009. *Morbidity and Mortality Weekly Report* 2009; **58**: 536–541.
12. **O'Riordan S, *et al.*** Risk factors and outcomes among children admitted to hospital with pandemic H1N1 influenza. *Canadian Medical Association Journal* 2009; **182**: 39–44.
13. **CDC.** Surveillance for pediatric deaths associated with 2009 pandemic influenza A (H1N1) virus infection – United States, April–August 2009. *Morbidity and Mortality Weekly Report* 2009; **58**: 941–947.
14. **CDC.** Swine influenza A (H1N1) infection in two children – Southern California, March–April 2009. *Morbidity and Mortality Weekly Report* 2009; **58**: 400–402.
15. **Simonsen L, *et al.*** Pandemic versus epidemic influenza mortality: a pattern of changing age distribution. *Journal of Infectious Diseases* 1998; **178**: 53–60.
16. **Baker M, Kelly H, Wilson N.** Pandemic H1N1 influenza lessons from the southern hemisphere. *Eurosurveillance* 2009; **14**(42): pii = 19370.
17. **WHO.** Recommended composition of influenza virus vaccines for use in the 2010 influenza season, 2009.
18. **Gates P, *et al.*** Collection of routine national seasonal influenza vaccine coverage data from GP practices in England using a web-based collection system. *Vaccine* 2009; **27**: 6669–6677.
19. **Office for National Statistics.** United Kingdom. Birth Statistics 2008. Series FMI No. 37. 9 February 2010 (http://www.statistics.gov.uk/downloads/theme_population/FMI-37/FMI_37_2008.pdf).
20. **Office for National Statistics.** Population estimates mid-2007 UK, England and Wales, Scotland and Northern Ireland, 2008.
21. **Health Protection Agency.** Method used to estimate new pandemic (H1N1) 2009 influenza cases in England in the week 3 August to 9 August 2009, 2009.
22. **'t Klooster TM, *et al.*** Surveillance of hospitalisations for 2009 pandemic influenza A(H1N1) in the Netherlands, 5 June–1 December 2009. *Eurosurveillance* 2010; **15**(2): pii = 19461.
23. **Cullen G, *et al.*** Surveillance of the first 205 confirmed hospitalised cases of pandemic H1N1 influenza in Ireland, 28 April–3 October 2009. *Eurosurveillance* 2009; **14**(44): pii = 19389.

24. **Echevarria-Zuno S, *et al.*** Infection and death from influenza A H1N1 virus in Mexico: a retrospective analysis. *Lancet* 2009; **374**: 2072–2079.
25. **Oliveira W, *et al.*** Pandemic H1N1 influenza in Brazil: analysis of the first 34,506 notified cases of influenza-like illness with severe acute respiratory infection (SARI). *Eurosurveillance* 2009; **14**(42): pii = 19362.
26. **CDC.** CDC Estimates of 2009 H1N1 influenza cases, hospitalizations and deaths in the United States, April 2009–January 16, 2010 Centres for Disease Control and Prevention, 2010.
27. **Luk J, Gross P, Thompson WW.** Observations on mortality during the 1918 influenza pandemic. *Clinical Infectious Diseases* 2001; **33**: 1375–1378.
28. **CDC.** Serum cross-reactive antibody response to a novel influenza A (H1N1) virus after vaccination with seasonal influenza vaccine. *Morbidity and Mortality Weekly Report* 2009; **58**: 521–524.
29. **Fuhrman C, *et al.*** Severe hospitalised 2009 pandemic influenza A(H1N1) cases in France, 1 July-15 November 2009. *Eurosurveillance* 2010; **15**(2): pii = 19463.
